# Can Instagram be used to deliver an evidence-based exercise program for young women? A process evaluation

**DOI:** 10.1186/s12889-020-09563-y

**Published:** 2020-10-06

**Authors:** Rachel G. Curtis, Jillian C. Ryan, Sarah M. Edney, Carol A. Maher

**Affiliations:** 1 Alliance for Research in Exercise, Nutrition and Activity, UniSA Allied Health and Human Performance, University of South Australia, GPO Box 2471, Adelaide, South Australia 5001 Australia; 2 Precision Health Future Science Platform, Commonwealth Scientific and Industrial Research Organisation, Adelaide, 5000 Australia; 3 Physical Activity & Nutrition Determinants in Asia, Saw Swee Hock School of Public Health, National University of Singapore, Singapore, 117549 Singapore

**Keywords:** Physical activity, Exercise, Instagram, Social media, Mhealth, Intervention

## Abstract

**Background:**

Instagram provides an opportunity to deliver low cost, accessible and appealing physical activity content. This study evaluated the feasibility of delivering an exercise program for young women using Instagram.

**Methods:**

A single-group pre- and post-intervention trial examined the feasibility and preliminary efficacy of a 12-week Instagram-delivered program with young inactive women (*n* = 16; *M* = 23 years), which prescribed running and body weight exercises to complete three times per week. Daily Instagram posts delivered the exercises, video demonstrations and motivational content. Feasibility was evaluated by examining exposure (Instagram posts viewed per week), engagement (likes, comments and tags on Instagram posts; number of exercise sessions completed per week; retention, defined as completion of the online survey at weeks 6 and 12), and acceptability [whether the program increased participants’ motivation to exercise (1 = strongly disagree-5 = strongly agree); satisfaction with the program (1 = not satisfied-5 = very satisfied)]. Preliminary efficacy was evaluated by comparing baseline and 12-week self-reported physical activity (IPAQ short-form) and fitness (cardiorespiratory and muscle strength; 1 = very poor-5 = very good, International Fitness Scale) using the Exact sign test.

**Results:**

On average, participants reported seeing six posts in their Instagram feed per week. Posts received an average of five likes (IQR = 3–6). A total of four comments and one tag were observed across all posts. On average, participants reported completing two exercise sessions per week. Retention was 88% at 6 weeks but dropped to 56% at 12 weeks. Participants reported increased motivation to exercise (Mdn = 4, IQR = 3–4) and were satisfied with the program (Mdn = 4, IQR = 3–4). Only self-reported cardiorespiratory fitness showed a meaningful, though nonsignificant, improvement (MdnΔ = 1, IQR = 0–1, *p* = .06).

**Conclusions:**

Although Instagram has the potential to deliver a low cost, convenient exercise program for young women, additional research is needed to identify methods of improving engagement (interaction with the Instagram content, exercise sessions completed, and retention in the program). Future research could examine the use of behaviour change theory and provide information that enables participants to tailor the exercises to their interests and needs. Additionally, the use of objective assessments of physical activity and fitness among a larger participants sample is needed.

## Introduction

Insufficient physical activity is a leading risk factor for non-communicable disease and premature death worldwide [1, 2]. Physical inactivity is estimated to cost Australia over $800 million per year in direct (e.g., healthcare) and indirect (e.g., lost productivity) costs [3]. Young Australian women are insufficiently active, with only 21% meeting recommendations for both moderate-to-vigorous activity (150 min per week) and muscle strengthening activities (2 days per week) [4]. It is therefore vital for young women to have access to appropriate resources and programs to help them take proactive steps to improve their physical activity and health. Evidence-based programs are needed that use low-cost, scalable methods of delivery and are therefore able to be widely disseminated and freely accessed by users. In addition, programs need to be delivered using methods that are appealing, relevant, and motivate sustained engagement over time.

Online social media such as Facebook, Instagram, and Twitter is extremely popular, particularly amongst young adults. For example, almost 8 in 10 Australians report using social media, with rates of use among those aged 18 to 29 years at 99% [5]. Social media provides an opportunity to offer health promotion programs in an engaging, interactive format to a large number of users at low cost. As well as enabling dissemination of health information, the features in social media (e.g., connecting with friends, commenting, liking and sharing posts) have the potential to increase motivation and facilitate ongoing engagement [6]. To date, health promotion research has examined Facebook and Twitter as delivery platforms [6–9] but the rising popularity of alternative social media platforms offers new possibilities for delivering appealing, socially-relevant health promotion programs. In particular, in 2017, 81% of Australian adults aged 18 to 29 used Instagram, and the typical user reported logging on 6 times per day [5]. Instagram therefore provides a unique opportunity to facilitate ongoing engagement with health promotion material throughout the day, maximising the potential for positive change in health behaviour.

Celebrity fitness pages are popular on Instagram, with some hosting millions of followers. However, free content is often limited to individual workouts and lifestyle and motivational posts, with users required to pay a fee or sign up to an ongoing paid subscription to receive the full exercise program. Additionally, celebrity programs may not be based on the best available evidence and may not be effective and safe long term. Preventive health approaches could capitalise the popularity of such pages to provide appealing, evidence-based physical activity programs that could be delivered at low cost and freely accessed by users. Despite the potential to use Instagram to provide health programs to young adults, there is a dearth of research in this area. Previous quasi-experimental research has examined whether Saudi female college students would adhere better to a home exercise program delivered via a YouTube video if they were also provided with an Instagram page that delivered information about the benefits of exercise and reminders to exercise [10]. Adherence (completing the exercise twice per week for 4 weeks) was poor in both intervention groups; 4% in the group that received the YouTube video only and 17% in the group that also received the Instagram page [10]. To our knowledge, no research has implemented an exercise program delivered entirely on Instagram. The aim of this research was therefore to conduct a process evaluation assessing the feasibility of a 12-week Instagram-delivered exercise program for young women, examining exposure, engagement, and acceptability (primary aim), and to assess the preliminary efficacy of the program, examining self-reported physical activity and fitness (secondary aim).

## Methods

Ethics approval was provided by University of South Australia Human Research Ethics Committee. Participants provided written consent prior to enrolling in the study.

### Program development

The program and Instagram content were informed by three formative focus groups conducted with a convenience sample of 13 women aged 19 to 30, recruited through word of mouth, who were insufficiently active (self-reported less than 150 min of physical activity per week). Participants were asked a series of structured questions about the types of physical activity they would like to see in an Instagram-based program, and the types of Instagram posts they would find motivating. Three key messages from the focus groups informed the program: (1) running and body weight exercises would be preferred because they could be performed outdoors without requiring equipment; (2) Instagram posts should feature people with average body types, rather than fitness models; (3) simple infographics and inspirational quotes should be included.

The 12-week program was developed in consultation with a clinical exercise physiologist (see Supplementary Table 1). It prescribed graded exercises to be completed 3 times per week in a 30 to 45-min moderate-intensity session at the participants’ preferred time and location. Each session consisted of a running prescription and a selection of body weight exercises such as sit-ups, lunges, squats and planks. The exercises began at a beginner level, targeted at those with a low level of fitness who were not previously exercising. Exercises became progressively more difficult each week in order to build participants’ fitness, except for weeks 4 and 8, which prescribed easier ‘deload’ exercise sessions in order to reduce fatigue and improve training adaptation [11].

Instagram posts were created using existing and purpose-designed content. One post per week outlined the exercises for the week. Other posts included video demonstrations of any exercises that were new that week (a selection of videos already publicly available on Instagram), motivational quotes (1–3 posts per week, e.g., “It’s not about having time. It’s about making time”), and informational posts (2–4 posts per week, e.g., the benefits of exercise; how to stretch). Each post included a caption (e.g., “Proper technique is a key to success”; “Tag a friend who has done a great workout this week”).

### Participants

Eligibility criteria were female, aged 18 to 30 years, insufficiently physically active (respond “no” to the question “do you do more than 150 minutes of physical activity per week?”), and currently use Instagram. Participants were a convenience sample from the greater Adelaide Metropolitan Region, who were invited word of mouth and snowball sampling to participate in a 12-week exercise program delivered via Instagram (July to August 2018). A sample size of 16 was considered sufficient to meet the primary aim of assessing feasibility of delivering an exercise program via Instagram [12]. Participants completed stage 1 of the Adult Exercise Pre-Screening Tool [13] prior to the commencement of the study.

### Procedure

The study used a single-group pre- and post-intervention design. Participants joined the purpose-designed Instagram page entitled “Thrive”, which was set to private so that participants could remain anonymous to Instagram users who weren’t enrolled in the study. Participants were encouraged to turn on page notifications. Daily posts delivered the intervention content. The page delivered one post per day: the weekly exercise program on Sundays, video demonstrations of new exercises on Mondays, and motivational and informational content on other days. The posts were uploaded to scheduling software Later (Later Media, Vancouver, Canada) prior to the commencement of the program and were automatically delivered for the duration of the program (August to November 2018). All participants began the intervention on the same day and received the same Instagram content.

Participants undertook a series of telephone support calls of approximately 10 min duration aimed at identifying any feasibility issues and providing troubleshooting assistance (e.g., ensuring participants saw the posts in their Instagram feed and could access and navigate the Instagram page). Calls were initially weekly (weeks 1, 2, and 3), then bi-weekly (weeks 5 and 7) to reduce participant burden. Calls ceased at week 7 because responses had not indicated any issues with the program content or delivery that required addressing. Participants completed an online survey at baseline, 6 weeks (during the intervention) and 12 weeks (post-intervention; see Supplementary Table 2 for survey items).

### Measures

#### Demographics and social media use

Participants reported their age, highest level of education [partial completion of high school, completion of Year 12, currently enrolled in vocational education and training (e.g., non-university certificate or diploma, including trade), completion of vocational education and training qualification, currently enrolled in a university degree, completion of a university degree], which social media platforms they used (Instagram, Facebook, Twitter, Other), and how often they used Instagram (never/less than once a month, 1–3 times per month, 1–6 times per week, 1–2 times per day, 3–6 times per day, 7+ times per day).

#### Feasibility

##### Exposure

Participants reported the number of posts seen in the past week via the telephone support calls.

##### Engagement

The number of likes, comments and tags on each Instagram post were examined. Participants reported the number of exercise sessions completed in the past week via the telephone calls. Retention was defined as completion of the online survey at weeks 6 and 12.

##### Acceptability

In the 12-week online survey, participants reported whether participating in the program had increased their motivation to exercise (1 = strongly disagree to 5 = strongly agree) and their overall satisfaction with the program (1 = not satisfied to 5 = very satisfied), using purpose designed items. Participants also provided open-ended responses to the items “What were the strengths of Thrive, in your opinion?” and “What were areas of improvement for Thrive, in your opinion?”

#### Preliminary efficacy

##### Physical activity

Participants reported the number of days and average time per day (minutes) they spent doing vigorous physical activity, moderate physical activity and walking, using the International Physical Activity Questionnaire short form (IPAQ-s), which is freely available to use without license [14]. Weekly minutes were calculated for each activity as days*minutes (with minutes in each domain truncated to 180 mins per day, as per recommendations [15]). Total MET-minutes/week was calculated as (vigorous days*mins*8.0) + (moderate days*mins*4.0) + (walking days*mins*3.3). The IPAQ-s has shown good test-retest reliability (pooled ρ = 0.76) and moderate agreement with activity measured via accelerometer (pooled ρ = 0.30) [14]. Participants reported the number of days and average time per day spent doing strengthening exercises using self-reported items created to match the format of the IPAQ-s (During the last 7 days, on how many days did you do strengthening exercises like push ups or weights at the gym? How much time (minutes) did you usually spend doing strengthening exercises on one of those days?). Weekly minutes of strengthening exercise was calculated as days*minutes.

##### Physical fitness

Participants rated their cardiorespiratory fitness and muscle strength relative to others their age using two items from the International Fitness Scale (IFIS), which is freely available to use [16]. Participants rated their cardiorespiratory fitness (capacity to do exercise, for example, running a long distance) and muscle strength, relative to others their age, on a scale on a scale from 1 = very poor to 5 = very good. The IFIS has shown moderate test-retest reliability (Kappa ranged from 0.54 to 0.65) and significant associations with objective measures of cardiorespiratory fitness and muscle strength in university students aged 18–30 years (*p* < .001 for all) [16].

### Analysis

#### Feasibility

##### Exposure

The number of Instagram posts viewed per week is presented descriptively using medians and interquartile ranges.

##### Engagement

The average number of likes per Instagram post is presented descriptively using a median and interquartile range. The overall number of comments and tags were summed. The number of exercise sessions completed per week is presented descriptively using medians and interquartile ranges. Retention was defined as the percentage of participants who completed the online survey at weeks 6 and 12.

##### Acceptability

Ratings for satisfaction and motivation are presented descriptively using medians and interquartile ranges. Open-ended responses for study strengths and recommended improvements were analysed using thematic analysis to identify common themes and are summarised as the number of participants that endorsed each reported strength or improvement.

#### Preliminary efficacy

Twelve-week change in physical activity and fitness variables were examined using the Exact sign test (rather than Wilcoxon signed rank test, as the distribution of differences was not symmetrical) in SPSS 25.

## Results

Participants were 16 females aged 18 to 28 years who reported regularly using Instagram (see Table 1).

**Table 1:** Participant characteristics (*n* = 16)

Characteristic	
Age (years), *M* (*SD*)	23 (3)
Education, n (%)	
Partial completion of high school	0
Completion of Year 12	4 (25)
Currently enrolled in vocational education and training	1 (6)
Completion of vocational education and training qualification	0
Currently enrolled in a university degree	8 (50)
Completion of a university degree	3 (19)
Social media use, n (%)	
Instagram	16 (100)
Facebook	16 (100)
Twitter	4 (25)
Other:	9 (56)
Instagram frequency, n (%)	
Never/Less than once a month	0
1–3 times per month	0
1–6 times per week	3 (19)
1–2 times per day	1 (6)
3–6 times per day	5 (31)
7+ times per day	7 (44)

### Feasibility

#### Exposure

On average, participants reported seeing 6 out of the 7 posts per week in their Instagram feed, which was relatively stable across weeks (week 1 Mdn = 6 IQR = 6–7, week 2 Mdn = 5 IQR = 4–6, week 3 Mdn = 6 IQR = 4–7, week 5 Mdn = 6 IQR = 3–6, week 7 Mdn = 6 IQR = 4–6).

#### Engagement

**Fig. 1:**
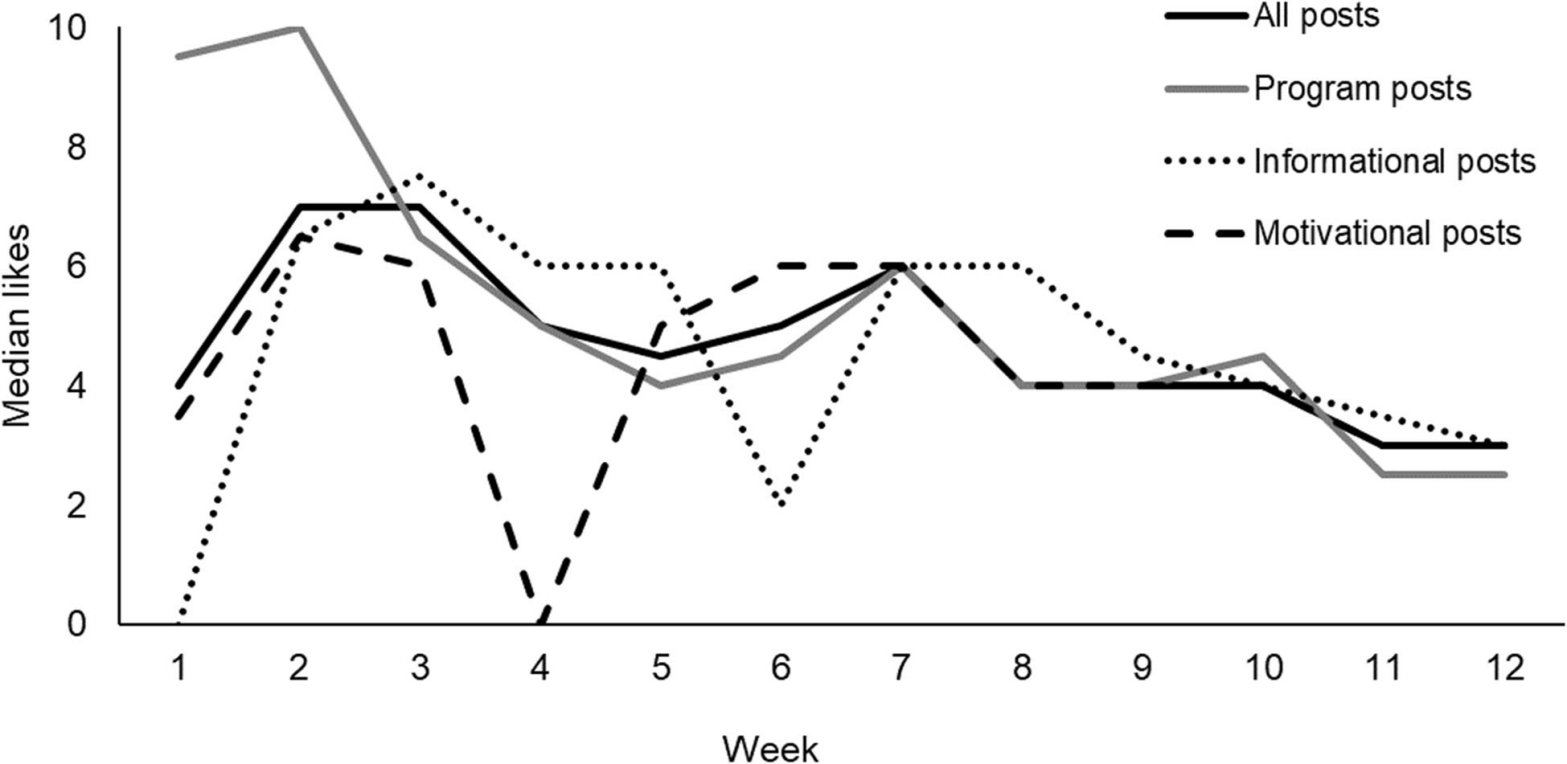
Median number of likes per Instagram post for each week of Thrive. *Note*: program posts include program information, exercise prescription and exercise demonstration videos. All types of posts (program posts, informational posts, motivational posts) were included between 1 and 4 times per week

Daily posts received an average (median) of 5 likes (IQR = 3–6) across the 12-week period. Likes were highest in weeks 2 and 3 and declined over the study period (see Fig. 1). There was a total of 4 comments and 1 tag across all posts. On average, participants reported completing 2 exercise sessions per week, which declined slightly over time (week 1 Mdn = 3 IQR = 2–3, week 2 Mdn = 2 IQR = 2–3, week 3 Mdn = 2 IQR = 1–3, week 5 Mdn = 2 IQR = 1.25–3, week 7 Mdn = 2 IQR = 0.25–2). Retention was high at 6 weeks (88%) but dropped at 12 weeks (56%).

#### Acceptability

At week 12, participants generally agreed that the program increased their motivation to exercise (median rating = 4, IQR = 3–4) and were satisfied with the program (median = 4, IQR = 3–4).

All participants who completed the week 12 survey (*n* = 9) provided open-ended responses, with some participants identifying multiple strengths (median = 2 strengths per participant, range = 1–3) or suggestions for improvement (median = 1, range = 1–2). Responses were reasonably brief for both strengths (median words = 5, range = 1–20) and suggestions for improvement (median words = 6, range = 1–35). Themes were reflected in four main categories: 1) strengths of the Instagram content, 2) strengths of the exercise prescription, 3) criticism and suggestions for the Instagram content, and 4) criticism and suggestions for the exercise prescription. The most common strengths of the Instagram content were that it was easy to access (*n* = 3), informative (*n* = 3) and motivating (*n* = 3; e.g., “Easy to follow, useful posts which were both motivational and informative”). The most common strength of the exercise prescription was that the progression of difficulty of the exercises was appropriate (*n* = 3; e.g., “Good amount of progression each week”). The most common criticism of the Instagram content was that it was repetitive (*n* = 2; e.g., “Posts were a little repetitive after a while”). The most common recommendation of the exercise prescription was to reduce the amount of running (*n* = 4; e.g., “In the latter weeks, don’t increase the running by as much”) and the most common criticism was that the exercises were repetitive (*n* = 3; e.g., “Repetitive exercises got a bit boring”). Supplementary Table 3 summarizes all identified themes and number of times each theme was identified.

### Preliminary efficacy

Table 2 displays self-reported physical activity and fitness at baseline, 6 weeks and 12 weeks. At baseline, most participants displayed low levels of physical activity (low *n* = 10, <600 MET-mins/week; moderate *n* = 4, 600–2999 MET-mins/week; high *n* = 2, ≥3000 MET mins/week) [15]. There were no significant changes in any of the physical activity measures at 12 weeks. At baseline, participants tended to rate their muscle strength and cardiorespiratory fitness as poor or average relative to others. Self-reported muscle strength was not improved at 12 weeks. At 12 weeks, participants tended to rate their cardiorespiratory fitness as average to good, suggesting a meaningful, though non-significant, improvement.

**Table 2:** Self-report physical activity and fitness

	Baseline *n* = 16		6 weeks^*a*^ *n* = 14		12 weeks^*b*^ *n* = 9		12-week change *n* = 9		
	Mdn	(IQR)	Mdn	(IQR)	Mdn	(IQR)	Mdn	(IQR)	*p*^*c*^
*IPAQ-s mins/week*									
Vigorous activity	0	(0–39)	0	(0–30)	0	(0–60)	0	(−23–30)	1.00
Moderate activity	20	(0–56)	30	(0–90)	60	(0–180)	60	(−45–170)	.43
Walking	120	(43–359)	125	(85–195)	120	(73–195)	−30	(− 265–43)	.73
Total MET-mins^*d*^	529	(289–2040)	714	(401–1353)	918	(541–1521)	297	(− 1197–671)	.51
Strengthening exercises	0	(0–33)	0	(0–30)	0	(0–35)	0	(−3–35)	1.00
*IFIS Fitness*									
Cardiorespiratory	2	(2–3)	3	(2–3)	3	(3–4)	1	(0–1)	.06
Strength	2	(2–3)	3	(2–3)	3	(3–3)	0	(− 0.5–1.5)	1.00

^*a*^During intervention, ^*b*^Post-intervention, ^*c*^Exact sign test (2-tailed), ^*d*^MET-minutes/week calculated as (vigorous days*mins*8.0) + (moderate days*mins*4.0) + (walking days*mins*3.3)

## Discussion

This novel study was the first to design and evaluate an exercise program for young women delivered entirely via Instagram. It was feasible to deliver an exercise program on Instagram, however, engagement with the program was modest. Additionally, there were trends for improvement in self-reported cardiorespiratory fitness, but not self-reported strength or self-reported physical activity. Results highlight some avenues for improving engagement with and efficacy of future exercise programs delivered via Instagram.

### Feasibility

Results suggest that an exercise program can be delivered to young women on Instagram using a cost-effective hands-off approach. Instagram content was uploaded to scheduling software Later, which delivered the Instagram posts each day with no further action from the research team. Although smartphone apps have become a popular method of disseminating health programs and have been shown to be moderately effective in increasing physical activity [17, 18], they can be expensive to develop. In contrast, Instagram is free to use, and programs can be created with a small budget by utilising content that is already on Instagram (e.g., in this study, Thrive shared existing videos of exercise demonstrations from fitness trainers). Additionally, in-person health programs incur significant costs per participant due to the reliance on research staff or practitioners to deliver the intervention, but health programs on Instagram are scalable at no added cost. The cost of delivering a program on Instagram is not dependant on the number of users; the cost of the scheduling software is determined by the number of social media pages and the number of scheduled posts per month (the cost to use Later for this study was $9USD per month).

Similar to the use of algorithms to determine content in a user’s Facebook feed [19, 20], Instagram uses proprietary algorithms and machine learning to determine which posts appear first in a user’s feed, based on their previous usage [21, 22]. A risk of using these social media platforms for health promotion programs is that researchers are unable to guarantee that participants will see their posts. If participants do not see the single weekly post with the exercise prescription, they would be required to navigate to the program page, which may affect acceptability and compliance. In this study, participants were encouraged to turn on notifications for the page and reported that they saw six posts in their feed most weeks. This appears promising, though, as objective data on post views was not obtained, we do not know whether participants were seeing the most important posts (i.e. the exercises).

Participants reported completing two out of the three prescribed sessions per week on average. Over 40% of participants dropped out of the program by week 12. Additionally, engagement with the Instagram content was low, with approximately 30% of participants liking each post, and most participants never commenting or tagging. Maintaining participant interest over time has been a major challenge for internet-delivered health interventions (see [23] for discussion). Many (though not all) interventions using social media and mobile phone apps to improve health behaviour have reported high rates of attrition and/or low engagement [9, 24, 25]. Research examining the efficacy of interventions that use mobile phone apps to improve diet, physical activity and sedentary behaviour suggests that higher app usage is associated with greater improvements physical activity and diet [18]. This is likely to apply to Instagram-based interventions, therefore future research should examine methods of maximising interaction with the program content.

During the telephone support calls, one participant mentioned that she didn’t feel comfortable commenting on or liking the Instagram posts because she didn’t know the other participants. Another participant said she might comment on posts if she knew more people in the program. Similarly, one participant thought that her motivation to complete the exercises might increase if she was able to exercise with other people. There is clear evidence to suggest that social support is associated with increased physical activity [26, 27], and that online social support may reduce attrition in physical activity programs [28]. The finding that participants had few interactions with each other on the Instagram page, coupled with the above comments, suggests that social support from pre-existing friends and family may be more appropriate for this subgroup of young women than social support from unfamiliar people. One recommendation to improve engagement with future programs is to harness participants’ existing social networks by enrolling them in groups of friends or family members. Additionally, internet-based health behaviour interventions that are based on behaviour change theory, in particular the Theory of Planned Behaviour, have been found to be more effective than those that are not based on theory [29]. Future research could examine whether engagement, particularly in terms of exercise sessions completed per week, is improved by incorporating behaviour change theory into the intervention through Instagram posts specifically designed to target self-efficacy, or barriers and enabler of exercise.

Participants reported that the program was easy to access, informative and motivating, increased their motivation to exercise, and they were satisfied with the program. This suggests overall acceptability of the program, however, results also indicate some potential for improvement. In the final survey, a few participants commented that the progression of the running component was too difficult. If participants were not fully compliant to the program, they may not have completed enough exercise sessions to gain the expected level of fitness. One recommendation is to provide information to enable participants to personalise the exercise program. It may be useful to provide video- or text-based educational content to teach users how to adapt the exercises to their own ability. For example, participants who were having difficulty performing the running component may have benefited from information about how to shorten their stride or reduce their pace. Additionally, some participants reported that they found the exercise program repetitive. Future iterations of the program could provide options of body weight exercises targeting each muscle group, allowing participants to choose exercises that they find to be most enjoyable.

### Preliminary efficacy

Although participants reported completing the exercises twice per week on average, self-reported physical activity on the IPAQ-s did not increase from baseline to 12 weeks. Median change in physical activity was 297 MET-mins/week but we would not consider this a trend, given the proportion of participants who showed a decline in activity over time. It is possible that participants were completing the program in lieu of other activities they previously performed; however, most participants were classified as inactive according to the IPAQ-s at baseline and would not have participated in regular physical activity prior to joining the program. Interestingly, participants reported a median of 0 min of strengthening exercises per week at all time points. The survey item asked about “strengthening exercises like push-ups or weights at the gym” and has not previously been validated. It is possible that participants did not consider the body weight exercises prescribed in the program to be strengthening exercises when completing the questionnaire. This seems an unlikely explanation, however, because push-ups (which were an explicit example in the survey question) were included in 7 weeks of the program. We note that we were likely underpowered for these analyses. Only nine participants could be included in this analysis, and patterns of change may have been clearer in larger sample.

Participants also did not show statistically significant improvements in self-reported strength or cardiorespiratory fitness. The World Health Organisation recommends that, to improve cardiorespiratory and muscular fitness, adults aged 18–64 years should do at least 150 min of moderate-intensity aerobic physical activity or 75 min of vigorous-intensity aerobic physical activity per week, as well as muscle-strengthening activities on at least 2 days per week [30]. If participants did not complete the program 3 times per week, they may not have engaged in enough physical activity to produce noticeable benefits. Additionally, participants may have overestimated their cardiorespiratory fitness and strength at baseline but reduced their perception of their own abilities when they began to partake in challenging exercises. As with analyses for self-reported physical activity, analyses for self-reported fitness were based on only nine participants and a larger sample is needed to fully examine program efficacy. Notably, although non-significant, the improvements in self-reported cardiorespiratory fitness were arguably meaningful as participants tended to improve their ratings from poor or average at baseline to average or good at 12 weeks. Differences between ratings of very poor/poor (combined), average and very good/good (combined) have previously been found to be associated with significant differences in 20 m shuttle run performance (*p* < .05 for all Bonferroni-adjusted pairwise comparisons) [16].

### Limitations

To our knowledge, this study is the first to evaluate an exercise program delivered via Instagram. The program was developed in consultation with exercise experts and formative research with end users, and contrasts with current Instagram offerings (in terms of full free program, non-models); however, limitations must be noted. The pilot sample was small, highly educated, and non-representative. Additionally, two participants were classified as highly active according to the IPAQ-s at baseline, suggesting either the screening item did not accurately identify those participants as active or the participants overestimated their activity at baseline on the IPAQ-s. All measures were self-reported except for the examination of likes, comments and tags on the Instagram posts, which were observed on the Instagram page. Although the IFIS has shown significant associations with objective measures of fitness [16], objective measurement such as the shuttle run test at each timepoint would provide stronger evidence for change in fitness. Similarly, an objective assessment of physical activity such as accelerometry may better capture changes in physical activity. Furthermore, there were no a priori criteria for engagement. Future research should specify an acceptable level of engagement (i.e. post views per week, number of likes, comments or tags in Instagram posts) to assess feasibility.

Only 56% of participants completed the 12-week survey. This data should therefore be treated with caution. Results may have been different if all participants had completed the final questionnaire. Retention may have been low because 50% of participants were currently enrolled in University, and 12-week assessments coincided with end-of-semester examinations. A rigorous reminder or follow-up procedure should be employed in future to reduce missing data.

The video demonstrations used in the program included a variety of women in different settings (e.g., gym, home, outdoors). This may have influenced feelings of continuity and impacted participant engagement. Additionally, we selected videos by fitness trainers that provided detail about how to ensure the correct form while performing the exercises. Although no videos depicted ‘extreme’ body types (e.g., extreme thinness or a body-building physique), most had a body type that would be considered thinner than average. Therefore, we were not always able to follow the recommendation from the focus groups to depict average body types in the program. Future iterations of the program could overcome this limitation by purpose-designing the videos. It is also important to note that the program was run as a private Instagram group, for research ethics reasons. A major advantage of using social media for health promotion is the potential for health information to spread rapidly through online social networks. Although running the program as a private group maintained participants’ privacy, running the program as a public page would likely extend the reach of the program and improve effectiveness. Future research should aim to use a larger representative sample and objective measures of physical activity and fitness, and evaluate the reach of the program through a public, rather than private, Instagram page.

## Conclusion

This study showed there is potential to use Instagram to deliver an exercise program for young women, but further research and development is needed before the program would be suitable for delivery on a large scale. The program was feasible to provide; it was low-cost, accessible and provided participants with sufficient information to enable them to perform the exercises. Generally, participants were satisfied with the program. However, the program did not show feasibility in terms of promoting a high level of engagement (low engagement with the Instagram content, moderate completion of exercise program, high drop-out) or preliminary efficacy in terms of increasing physical activity and fitness. Future research should aim to identify methods of maximising engagement with physical activity content on Instagram, and examine how increased engagement affects exercise adherence. Engagement with the program might be improved through incorporating behaviour change theory and enabling participants to tailor the exercise program to their needs. The use of objective assessments of physical activity and fitness among a larger participants sample is also required to more accurately assess the efficacy of the program.

## Supplementary information

**Additional file 1: Table S1.** Thrive 12-week exercise program, to be completed three times per week.

**Additional file 2: Table S2.** Survey items. A list of all survey items that were used in this study, indicating response options and data collection timepoints.

**Additional file 3: Table S3.** Results from open-ended survey items at week 12.

## Data Availability

The datasets used and/or analysed during the current study are available from the corresponding author on reasonable request.

## Abbreviations

IPAQ-s: International Physical Activity Questionnaire short form
IFIS: International Fitness Scale
