# Supplementary material for: Can Instagram be used to deliver an evidence-based exercise program for young women? A process evaluation
Source: BMC Public Health. 2020 Oct 6;20:1506. doi: 10.1186/s12889-020-09563-y (PMC7539409; doi:10.1186/s12889-020-09563-y)
Supplement: Supplementary file 1 — Additional file 1: Table S1. Thrive 12-week exercise program, to be completed three times per week. [file 12889_2020_9563_MOESM1_ESM.docx]

**Supplementary Table 1.** Thrive 12-week exercise program, to be completed three times per week

| Week | Running prescription |  |  | Bodyweight prescription | | |
| --- | --- | --- | --- | --- | --- | --- |
|  | Exercise | Time | Instruction | Exercises | Sets | Reps^b^ |
| 1 | Comfortable jog Walking | 2 min 2 min | Alternate for 15 mins | Sit-ups Push-ups (on knees)  Star-jumps Lunges | 1 | 6 |
| 2 | Comfortable jog Walking | 2 min 1 min | Alternate for 15 mins | Sit-ups Push-ups (on knees) Star-jumps Lunges | 2 | 6 |
| 3 | Moderate-intensity run Walking | 2 min 1 min | Alternate for 15 mins | Sit-ups Push-ups (on knees) Star-jumps Donkey kicks | 2 | 7 |
| 4^a^ | Comfortable jog Walking | 2 min 1 min | Alternate for 15 mins | Sit-ups Push-ups (on knees) Star-jumps Donkey kicks | 2 | 6 |
| 5 | Moderate-intensity run Walking | 5 min 2 min | Alternate for 15 mins | Sit-ups Dips Squats Donkey kicks | 2 | 8 |
| 6 | Moderate-intensity run Walking | 6 min 1.5 min | Alternate for 15 mins | Bicycle sit-ups Dips Squats Donkey kicks | 3 | 8 |
| 7 | Moderate-intensity run Walking | 10 min 2 min | Repeat twice | Bicycle sit-ups Dips Squats Donkey kicks | 3 | 9 |
| 8 ^a^ | Moderate-intensity run Walking | 6 min 1.5 min | Repeat twice | Bicycle sit-ups Dips Lunges Donkey kicks | 2 | 8 |
| 9 | Moderate-intensity run Walking | 10 min 1 min | Repeat twice | Bicycle sit-ups Burpees Lunges Donkey kicks | 3 | 10 |
| 10 | Moderate-intensity run Walking | 15 min 1 min | Repeat twice | Plank (30 secs) Push-ups (on knees) Burpees Lunges | 3 | 12 |
| 11 | Moderate-intensity run Walking | 15 min 45 sec | Repeat twice | Plank (30 secs) Burpees Body-weight rows OR push-ups (knees off ground) Squat jumps | 3 | 13 |
| 12 | Moderate-intensity run Walking | 15 min 30 sec | Repeat twice | Plank (1 min) Burpees Body-weight rows OR push-ups (knees off ground) Squat jumps | 3 | 15 |

^a^ Weeks 4 and 8 are de-load weeks
^b^ Reps = repetitions for each exercise per set
