# Supplementary material for: Can Instagram be used to deliver an evidence-based exercise program for young women? A process evaluation
Source: BMC Public Health. 2020 Oct 6;20:1506. doi: 10.1186/s12889-020-09563-y (PMC7539409; doi:10.1186/s12889-020-09563-y)
Supplement: Supplementary file 2 — Additional file 2: Table S2. Survey items. A list of all survey items that were used in this study, indicating response options and data collection timepoints. [file 12889_2020_9563_MOESM2_ESM.docx]

Supplementary Table 2: Survey items

| **Item** | **Baseline** | **6 weeks** | **12 weeks** |
| --- | --- | --- | --- |
| What is your age? ___________________ | ✓ |  |  |
| What is the highest level of education that you have completed?   - Part of High School - Completion of Year 12 - Currently enrolled at University - Completed a University degree - Currently enrolled at TAFE (or similar) - Completed a TAFE (or similar) qualification - Postgraduate studies (e.g., Masters, PhD etc) | ✓ |  |  |
| What is your current occupation? ________________ | ✓ |  |  |
| Which social media platforms do you use?   - Instagram - Facebook - Twitter - Other: ______________________ | ✓ |  |  |
| How often do you use Instagram?   - Never/Less than once a month - 1-3 times per month - 1-6 times per week - 1-2 times per day - 3-6 times per day - 7+ times per day | ✓ |  |  |
| During the last 7 days, on how many days did you do strengthening exercises like push ups or weights at the gym? | ✓ | ✓ | ✓ |
| How much time (minutes) did you usually spend doing strengthening exercises on one of those days? | ✓ | ✓ | ✓ |
| International Physical Activity Questionnaire, short form (IPAQ-s) [1]  All items used. | ✓ | ✓ | ✓ |
| International Fitness Scale (IFIS) [2]  Cardiorespiratory fitness and strength items used. | ✓ | ✓ | ✓ |
| Thrive has helped increase your motivation to exercise.  1. Strongly disagree  2. Disagree  3. Neutral  4. Agree  5. Strongly agree |  |  | ✓ |
| Overall how satisfied were you with Thrive?  1. Not satisfied  2.  3.  4.  5. Very satisfied |  |  | ✓ |
| What were the strengths of Thrive, in your opinion? _____________ |  |  | ✓ |
| What were areas of improvement for Thrive, in your opinion? _____ |  |  | ✓ |

**References**

1. IPAQ Research Committee. Guidelines for the data processing and analysis of the "International Physical Activity Questionnaire". 2005. <https://sites.google.com/site/theipaq/scoring-protocol>. Accessed 19 Jul 2018.

2. Ortega FB, Sánchez-López M, Solera-Martínez M, Fernández-Sánchez A, Sjöström M, Martínez-Vizcaino V. Self-reported and measured cardiorespiratory fitness similarly predict cardiovascular disease risk in young adults. Scand J Med Sci Sports. 2013;23:749-57.
