# Supplementary material for: Can Instagram be used to deliver an evidence-based exercise program for young women? A process evaluation
Source: BMC Public Health. 2020 Oct 6;20:1506. doi: 10.1186/s12889-020-09563-y (PMC7539409; doi:10.1186/s12889-020-09563-y)
Supplement: Supplementary file 3 — Additional file 3: Table S3. Results from open-ended survey items at week 12. [file 12889_2020_9563_MOESM3_ESM.docx]

Supplementary Table 3. Results from open-ended survey items at week 12 (n=9)

| Category | Theme | n | Examples |
| --- | --- | --- | --- |
| Strengths of the Instagram content | Easy to access | 3 | “Easy to follow, useful posts which were both motivational and informative”  “Regular posts with relevant content”  “Posts made you feel accountable to complete the program” |
|  | Easy to understand | 2 |  |
|  | Informative | 3 |  |
|  | Motivating | 3 |  |
|  | Relevant | 1 |  |
|  | Regular | 1 |  |
|  | Promoted accountability | 1 |  |
| Strengths of the exercise prescription | Appropriate progression of difficulty | 3 | “Good amount of progression each week”  “Gradual increase in fitness” |
|  | Increased fitness | 1 |  |
|  | Varied exercises | 1 |  |
| Criticisms/suggestions for the Instagram content | Repetitive | 2 | “Post were a little repetitive after a while”  “Provide more engaging posts in the second half of the program - light encouragement was nice in the earlier stages however, as it went on it would have been nice for some deeper and more meaningful posts” |
|  | Recommend more engaging posts in the second half of the program | 1 |  |
|  | Recommend clearer demonstration videos | 1 |  |
|  | Recommend videos posted on Sundays | 1 |  |
| Criticisms/suggestions for the exercise prescription | Repetitive | 3 | “Repetitive exercises got a bit boring” |
|  | Recommend less running | 4 | “In the latter weeks, don't increase the running by as much” |
|  | Recommend more body weight exercises | 1 | “Less running, more floor exercises” |
